# Efficacy and safety of *Lacticaseibacillus paracasei* Lpc-37® in students facing examination stress: A randomized, triple-blind, placebo-controlled clinical trial (the ChillEx study)

**DOI:** 10.1016/j.bbih.2023.100673

**Published:** 2023-08-01

**Authors:** Sanna M. Mäkelä, Síle M. Griffin, Jenni Reimari, Kara C. Evans, Ashley A. Hibberd, Nicolas Yeung, Alvin Ibarra, Jouni Junnila, Jari Turunen, Ronnie Beboso, Balgit Chhokar, Timothy G. Dinan, John Cryan, Elaine Patterson

**Affiliations:** a IFF Health & Biosciences, Kantvik, Finland; b IFF Health & Biosciences, Madison, USA; c 4Pharma Ltd, Turku, Finland; d MeDiNova North London Dedicated Research Center, London, UK; e MeDiNova East London Dedicated Research Center, London, UK; f APC Microbiome Ireland, University College Cork, Cork, Ireland

**Keywords:** Psychological stress, *Lacticaseibacillus paracasei* Lpc-37, Anxiety, Probiotic

## Abstract

*Lacticaseibacillus paracasei* Lpc-37 (Lpc-37) has previously shown to reduce perceived stress in healthy adults. The ChillEx study investigated whether Lpc-37 reduces stress in a model of chronic examination stress in healthy students. One hundred ninety university students (18–40 y) were randomized to take 1.56 × 10^10^ colony-forming units of Lpc-37 or placebo (1:1) each day for 10 weeks, in a triple-blind, parallel, multicenter clinical trial consisting of six visits: two screening visits, a baseline visit, and visits at 4, 8, and 10 weeks after baseline. The primary objective was to demonstrate that Lpc-37 reduces stress, as measured by the change in state anxiety from baseline to just before the first examination, after 8 weeks using the State Trait Anxiety Inventory (STAI-state). Secondary objectives aimed to demonstrate that Lpc-37 modulates psychological stress-induced symptoms and biomarkers related to mood and sleep. An exploratory analysis of fecal microbiota composition was also conducted. There was no difference between Lpc-37 and placebo groups in the change of STAI-state score (estimate 1.03; 95% confidence interval [CI]: -1.62, 3.67; p = 0.446). None of the secondary outcomes resulted in significant results when corrected for multiplicity, but exploratory results were notable. Results showed an improvement in sleep-disturbance scores (odds ratio 0.30; 95% CI: 0.11, 0.82; p = 0.020) and reduction in duration of sleep (odds ratio 3.52; 95% CI: 1.46, 8.54; p = 0.005) on the Pittsburgh Sleep Quality Index questionnaire after 8 weeks in the Lpc-37 group compared to placebo. A reduction in Bond Lader VAS-alertness was also demonstrated in the Lpc-37 group compared to placebo (estimate −3.97; 95% CI: -7.78, −0.15; p = 0.042) just prior to the examination. Analysis of fecal microbiota found no differences between study groups for alpha and beta diversity or microbiota abundance. Adverse events were similar between groups. Vital signs, safety-related laboratory measures, and gastrointestinal parameters were stable during the trial. In conclusion, probiotic Lpc-37 was safe but had no effect on stress, mood, or anxiety in healthy university students in this model of chronic academic stress. ClinicalTrials.gov: NCT04125810.

## 1 Introduction

The role of the gut microbiome in regulating stress-related changes in physiology, behavior, and brain function is emerging (Foster et al., 2017). The microbiota-gut-brain axis modulates mood and behavior in animal models (Cryan et al., 2019) and possibly in humans, offering new opportunities for interventions targeting mental health with so-called psychobiotics, which are any exogenous influence whose positive effect on mental health and well-being are bacterially mediated, probiotics for example (Dinan et al., 2013; Sarkar et al., 2016). Stress exposure, in early life or adulthood, can alter the gut microbial profile to shape stress responsiveness and behavior (Vogel et al., 2020). Fecal microbiota transplantation studies in mice have shown that an anxious phenotype can be transplanted through the gut microbiome (Chinna Meyyappan et al., 2020), and even behavioral phenotypes of psychiatric disorders, such as major depressive disorder (MDD), can be transferred from human to rat via feces (Kelly et al., 2016), further emphasizing the role of the gut microbiome in mood. *Lacticaseibacillus rhamnosus* JB-1 has been shown to reduce anxiety and depressive-like behavior in rats (Chudzik et al., 2022). Hence, the gut microbiota composition can be altered through stressful life events, and the gut microbiota can have an impact on behavior and mental health.

Previous clinical trials have used an examination stress model to study whether probiotics affect stress-related outcomes in healthy students but have produced mixed results (Kato-Kataoka et al., 2016b; Marcos et al., 2004; Nishida et al., 2017; Takada et al., 2016, 2017). Fermented milk containing *Lactobacillus casei* Shirota reduced the incidence of cold/flu symptoms and abdominal symptoms, such as abdominal pain or discomfort, in healthy medical students undergoing university examinations (Takada et al., 2016). Moreover, in healthy college students, panic anxiety and neurophysiological anxiety were improved with administration of a multispecies probiotic (Tran et al., 2019). According to a meta-analysis, probiotics reduced subjective stress levels in healthy volunteers but did not influence cortisol levels (Zhang et al., 2020).

*Lacticaseibacillus paracasei* Lpc-37 (Lpc-37) has been shown to modulate anxiety and depression-related behavior in a mouse model of daily chronic restraint stress (Stenman et al., 2020). Moreover, supplementation with Lpc-37 improved recognition memory deficits in the novel-object recognition test, spatial working memory deficits in the Y-maze, and contextual long-term memory impairments in the step-through passive avoidance task in sleep-deprived mice (Griffin et al., 2022). In a double-blind, randomized, placebo-controlled clinical trial, Lpc-37 improved psychological and physiological markers of stress and anxiety (Patterson et al., 2020). The intake of Lpc-37 significantly reduced perceived stress compared with placebo, as assessed with Cohen's Perceived Stress Scale (PSS).

The aim of the current study was to investigate whether Lpc-37 modulates the psychological stress experienced by healthy medical, dental, and health science students preparing for university semester examinations. The primary outcome was assessed with the self-reported questionnaire, State Trait Anxiety Inventory (STAI) Form Y-1, that measures state anxiety, a transitory emotional state consisting of feelings of apprehension, nervousness, and physiological sequelae (Spielberger et al., 1983). Secondary outcomes were assessed using other inventories related to stress, anxiety, subjective feelings, and sleep.

## 2 Materials and methods

### 2.1 Ethics

The study was approved by ethics committees in United Kingdom and Ireland and conducted in accordance with ICH Guidelines on Good Clinical Practice (ICH Harmonised Guideline, 2015) and the Declaration of Helsinki (World Medical Association, 2013). Written informed consent was obtained from all participants. The study was registered at ClinicalTrials.gov: NCT04125810.

### 2.2 Study design

**Fig. 1:**
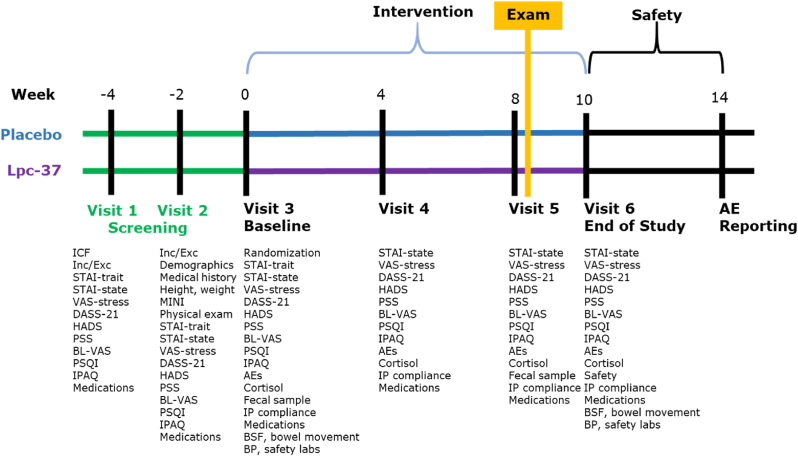
Study design. Abbreviations: AEs = adverse events; BL-VAS = Bond-Lader Visual Analogue Scale; BP = blood pressure; BSF = Bristol Stool Form; DASS-21 = Depression, Anxiety, and Stress Scales – 21 items; HADS = Hospital Anxiety and Depression Scale; ICF = informed consent form; Inc/Exc = inclusion and exclusion criteria; IP = investigational product; IPAQ = International Physical Activity Questionnaire; MINI = Mini International Neuropsychiatric Interview; PSQI = Pittsburgh Sleep Quality Index; PSS = Cohen's Perceived Stress Scale; STAI = State Trait Anxiety Inventory; VAS = Visual Analogue Scale.

The ChillEx study was a randomized, triple-blind, placebo-controlled, two-arm (parallel groups) clinical trial conducted at six sites in the United Kingdom and one in Ireland. The study design (Fig. 1) included two screening clinic visits (V1 and V2) approximately 4 weeks and 2 weeks prior to the randomization and the baseline visit (V3). Participants were assigned to intake either Lpc-37 or placebo over 10 weeks, with clinic visits at week 4 (V4); at week 8 (V5), just prior to the first semester examination; and at week 10 (V6), approximately 2 weeks after the first semester examination. The intervention duration of 8 weeks before the examination was chosen based on previous studies with 6–12 weeks of treatment (Kato-Kataoka et al., 2016b; Marcos et al., 2004; Nishida et al., 2017; Takada et al., 2016, 2017). In case of ongoing adverse event (AE), the participant was followed for 4 weeks after completion of the intervention.

At the screening visits participants were asked about their demographics, medical history, concomitant medications, physical activity, significant self-calming techniques, and life stressors. Participants were screened using the Mini International Neuropsychiatric Interview (MINI) to exclude those with any significant psychiatric diagnosis, according to the Diagnostic and Statistical Manual of Mental Disorders, 4th edition (DSM-IV). Race and ethnicity data were collected according to U.S. Food and Drug Administration guidelines. At screening V2, participants completed a battery of self-report scales and inventories. Fecal and saliva sampling kits and instructions were dispensed to the participants. Participants were given paper diaries to report AEs, concomitant medications, stool form on the Bristol Stool Form (BSF) scale, and frequency of bowel movements. Weight, height, and blood pressure were collected, and fasting blood and urine tests were performed.

The primary outcome was assessed at V5, just prior to semester examinations (Fig. 1). Saliva samples were collected at V3, V4, V5, and V6. All the questionnaires were completed at all visits, except the trait portion of the STAI, which was administered only at screening. Fecal samples were collected from about 50% of the participants in each group at V3 and V5.

### 2.3 Study participants

Participants were recruited from universities and institutions via targeted advertising. Recruited individuals were eligible to participate if they were enrolled in a medical, dental, or health science course at a university or institute of technology, intended to sit for the semester examination(s), were male or female 18–40 years old, and were generally healthy, with a BMI between 18.5 and 29.9 kg/m^2^. Individuals with mental disorders (e.g. depression, anxiety disorder, bipolar spectrum disorder or schizophrenia) or chronic illness (e.g. cardiovascular, gastrointestinal immunological, metabolic or neurodevelopmental disease), individuals on psychoactive medication, dietary supplements, or probiotics, and those with a recent history of antibiotic therapy were excluded. The full description of eligibility criteria is included in Supplementary Methods.

### 2.4 Investigational product (IP)

The IP was 1.56 × 10^10^ colony-forming units (CFU) of Lpc-37 with micro-crystalline cellulose, 1% magnesium stearate, and 1% silicon dioxide as carriers in one Capsugel V-Caps Size 1 hydroxypropyl methylcellulose (HPMC) capsule. The placebo product (micro-crystalline cellulose, 1% magnesium stearate, and 1% silicon dioxide) was identical to the Lpc-37 product in appearance and taste. The study products were manufactured by Danisco USA Inc. (Madison, USA) and distributed to randomized participants in bottles containing 40 (±1) capsules at V3, V4, and V5. Study participants were instructed to consume one capsule, with a glass of plain water, daily for approximately 10 weeks at any time of the day. Participant compliance was determined by counting the capsules returned by the participants.

### 2.5 Study outcomes

#### 2.5.1 Primary outcome: STAI-state

State anxiety was evaluated using the STAI Form Y-1. The STAI is a self-report inventory consisting of 2 sub-scales, each with 20 items. One sub-scale (Form Y-1), measures state anxiety, i.e., current state of anxiety; the other sub-scale (Form Y-2) measures trait anxiety, i.e. stable aspects of anxiety proneness (Spielberger et al., 1983). The primary outcome was calculated as change in state anxiety from V3 to V5. The change in state anxiety from V3 to V4 and V6 were evaluated as ancillary endpoints.

#### 2.5.2 Secondary outcomes

Secondary outcomes were to investigate the effect of Lpc-37 on psychological stress-induced symptoms and biomarkers, compared with placebo. Endpoints derived from measurements of cortisol concentrations from saliva (Cortisol awakening response (CAR)) were incremental area under curve (AUC_i_), AUC ground (AUC_g_), maximum concentration (C_max_), cortisol at awakening (timepoint zero), mean increase (C_max_ - cortisol at zero timepoint), and evening cortisol.

Perceived stress was measured using the visual analogue scale (VAS) where participants indicated along a 100-mm scale how stressed they perceived themselves to be. The Depression, Anxiety and Stress Scales – 21 items (DASS-21), was used to evaluate negative emotional states of depression, anxiety, and stress (Lovibond and Lovibond, 1995) during the past week. The 14-item self-reported Hospital Anxiety and Depression Scale (HADS) was used to measure levels of anxiety and depression within the last week.

Perception of stress was measured using Cohen's Perceived Stress Scale (PSS) (Cohen et al., 1983). The PSS asks 10 questions about feelings and thoughts during the last month; it evaluates the degree to which life situations are perceived as stressful, on a scale from 0 to 40, with higher scores indicating greater perceived stress.

Subjective feelings were measured using Bond-Lader (BL-VAS), which consists of 16 visual analogue scales with anchors of related mood or arousal factors (e.g., calm-excited, strong-feeble) (Bond and Lader, 1974). Participants indicated on a 100-mm line how they felt along each mood axis at that specific time. Scores from the 16 scales were converted into three composite mood/arousal factors: alertness, calmness, and contentedness.

Sleep was measured using the Pittsburgh Sleep Quality Index (PSQI), which assesses sleep quality over the prior month (Buysse et al., 1989) and includes 19 items measuring seven key components of sleep: sleep latency, sleep duration, sleep efficiency, sleep disturbances, subjective sleep quality, use of sleep medication, and daytime dysfunction due to sleepiness with minimum score of 0 (best) and maximum score of 3 (worst). Scores on each component were summed for a PSQI total score from 0 to 21. A total score of <5 indicates good sleep quality, and >5 indicates poor sleep quality.

#### 2.5.3 Ancillary and exploratory outcomes

One ancillary outcome was to demonstrate the effect of Lpc-37, compared with the placebo, on physical activity, measured using the International Physical Activity Questionnaire (IPAQ)-short (Craig et al., 2003). The study also explored the effect of Lpc-37, compared with placebo, on fecal microbiota composition and diversity. To confirm the compliance of study product, Lpc-37 was examined from feces of participants by qPCR.

#### 2.5.4 Safety and vital signs

All adverse events and concomitant diseases were recorded and coded using the Medical Dictionary for Regulatory Activities (MedDRA). Concomitant medications were coded using Anatomical Therapeutic Chemical (ATC) classification. Safety assessment at V2 and V6 included vital signs (blood pressure and heart rate) plasma aspartate transaminase (ASAT), alanine transaminase (ALAT), and gamma-glutamyl transferase (GGT); serum creatinine and urine sodium, potassium, and urea. Gastrointestinal safety parameters included self-reported evaluations of stool consistency (on the BSF) and bowel movement frequency during the week before V3 and V6.

### 2.6 Biological sample collection and analysis

#### 2.6.1 Saliva and fecal sample collection and storage

Participants collected saliva during two consecutive working days before V3, V4, V5, and V6 in Salivette® (Sarstedt, Nuembrecht, Germany) devices at 0, 30, and 45 min post-wakening (cortisol awakening response, CAR) and at 8 p.m. (evening cortisol), according to kit instructions. The samples were stored in the refrigerator at home until they were transferred to the study nurse at visits. Saliva samples were stored at < −20 °C at the clinic and sent for analysis to Daacro GmbH & Co., Germany. Participants collected fecal samples using Commode Specimen Collection System (BMP Medical, Sterling, USA) and 30 ml Para-Pak Clean Vials (Meridian Bioscience, Cincinnati, USA) at home before V3 and V5, stored them frozen, and brought them to the clinic in cooler bags. Fecal samples were stored at < −20 °C at the clinic until analysis.

#### 2.6.2 Salivary cortisol analysis

Salivary cortisol levels were determined with a high-sensitivity salivary cortisol competitive immunoassay kit (Salimetrics, State College, PA, United States) according to the manufacturer's instructions. All samples were analyzed in duplicate. After assay completion, optical density was read on a BioTek ELx808 microplate reader at 450 nm. Cortisol concentration was calculated using Gen 5 v. 3.08 (BioTek Instruments, Winooski, VT, United States) with a 4-parameter non-linear regression standard curve. Assay quality was measured by calculating the intra-assay coefficient of variation (CV) and inter-assay variability, which were 2.63% and 3.38%, respectively. Samples with higher values than the highest standard were diluted and re-analyzed. The upper limit of quantification was 82.77 nmol/l, and the lower limit was 0.19 nmol/l.

#### 2.6.3 DNA isolation and qPCR

Fecal sample DNA was isolated as described (Mukerji et al., 2016). Briefly, 200 mg of feces were lysed using Precellys 24 Homogenizer (Bertin Instruments, Montingy-le-Bretonneuz, France). Nucleic acid was extracted from the lysate using the Applied Biosystem AM1840 MagMAX™ Total Nucleic Acid Isolation kit on the MagMAX™ Express 96 DNA extraction robot (Thermo Fisher Scientific, Waltham, MA, United States) and purified using the OneStep-96 PCR Inhibitor removal kit (Zymo Research, Irvine, CA, United States). DNA was quantified using the Qubit 3.0 Fluorimeter and Qubit dsDNA HS kit (Thermo Fisher Scientific, Waltham, MA, United States).

Lpc-37 DNA was analyzed by qPCR with strain-specific primers and a probe targeting a unique region within a CRISPR repeat (forward primer 5’-TTGGGTGCTATGGGAAACACA, reverse primer 5’-AAGTTACGCCGCCACAAAC, probe 5’-GCGTGATTCAGGACATTCTGGACGAAGGAC). The assays were run using 400 nM forward and reverse primer and 200 nM probe in a total reaction volume of 25 μl. The reactions were conducted in triplicate using Taqman FAST Advanced MasterMix and the QuantStudio 5 Real Time PCR System (Applied Biosystems, Waltham, MA, United States) according to manufacturer's instructions.

#### 2.6.4 16S sequencing of fecal microbiota

The microbiota from fecal samples were assessed by 16S amplicon sequencing as previously described (Caporaso et al., 2011). Briefly, the 16S rRNA fourth hypervariable region was amplified in triplicate using barcoded primers 515F (5’-GTGCCAGCMGCCGCGGTAA) and 806R (5’-GGACTACHVGGGTWTCTAAT). PCR products were purified, normalized by DNA concentration, and pooled into 3 libraries for sequencing with 2 replicate MiSeq v2 (2 × 250 bp) runs at the W.M. Keck Center at University of Illinois–Urban, USA. See the sequencing data analyzes details in the Supplementary Methods.

### 2.7 Statistics

#### 2.7.1 Sample size calculation

The sample size was calculated assuming 90% power to reject the null hypothesis (no difference between the Lpc-37 group and placebo group in the change from baseline in STAI-state total score) with a 5% level of significance. Assumed effect size was based on a clinically meaningful difference of 6 points in STAI-state total score between the placebo and Lpc-37 groups. A standard deviation (SD) of 12 was selected as most plausible for the change from baseline in STAI-state total score (Marcos et al., 2004). The expected drop-out rate was approximately 10%, so that a recruitment of 190 participants would result in 172 evaluable participants. Power calculations were made using NQuery Advisor v. 8.2 (Statistical Solutions Ltd, Cork, Ireland).

#### 2.7.2 Randomization and blinding

Participants were randomized by computer to the Lpc-37 or placebo group in a 1:1 ratio using block randomization stratified by gender. The randomization was performed in an electronic data capture system Viedoc™ (Viedoc Technologies AB, Uppsala, Sweden) by the investigator or delegate and was managed by Oy 4Pharma Ltd (Turku, Finland). All study site personnel, contract research organization personnel, and sponsor representatives responsible for the study management or sample analysis were kept blinded throughout the study. The blind was broken only after all data were recorded and verified, the database was locked, and the statistical analysis plan (SAP) was completed. Statistical reporting was conducted using a partly blinded database that revealed only group allocation (A versus B). The investigators were provided with an electronic unblinding function in Viedoc™ to break the code for a participant in case of emergency.

#### 2.7.3 Statistical methods

Intention to treat (ITT) was the primary population of the study, except for the fecal microbiota analysis (see section 2.6.3). One site with only four participants was pooled with another site on geographical grounds. A linear mixed model with repeated measures (MMRM) was fitted for the primary efficacy variable. The model included the following fixed effects: baseline STAI-state total-score, sex, pooled study site number, treatment group, visit (V4, V5, V6) as a repeated factor, and the interaction between treatment group and visit. Participant served as the random effect in the model. The contrast between the Lpc-37 group and the placebo group at V5 was estimated from the model with a 95% confidence interval (CI). The corresponding two-sided p-value for the null hypothesis was obtained from the model and used in the evaluation of the superiority hypothesis.

Subgroup analyses were conducted for sex and for high (>30) versus low (≤30) baseline STAI-trait score, using the median baseline score of 30 as the cutoff. Models for the subgroup analyses were similar to models used for the primary analysis, with the addition of the subgroup term and subgroup-by-treatment interaction. The subgroup-by-treatment interaction was dropped if it was not significant.

The 24 secondary endpoints in the study were analyzed hierarchically using a fixed sequence procedure. (The hierarchy was predefined in the SAP, as described in the Supplementary Methods.) Testing continued until the first null hypothesis was accepted. The p-values for all secondary comparisons are shown, but those calculated after the acceptance of first null hypothesis are considered exploratory. The secondary outcomes [change from baseline to V5 in AUC_i_ (CAR), C_max_ of cortisol, cortisol at awakening, evening cortisol, VAS-stress score, DASS-21 stress score, HADS anxiety score, PSS total score, BL-VAS alertness score, BL-VAS contentment score, BL-VAS calmness score, PSQI total score, AUC_g_ (CAR), and cortisol mean increase] were analyzed with MMRM models similar to those used for the primary outcome. The cortisol AUC areas under the concentration-time curve from time zero to 45 min were calculated with the linear trapezoidal rule method. The seven component scores of the PSQI, the DASS-21 depression score, DASS-21 anxiety scores, and HADS depression scores were analyzed with cumulative logit models. For DASS-21 stress and anxiety scores, four categories were defined based on data: change less than zero, equal to zero, greater than zero but less than five and change greater than five. For HADS depression score, three categories were used: change less than zero, equal to zero, and greater than zero.

The ancillary efficacy endpoints of the study were change in physical activity level from baseline to V4–V6, detection of Lpc-37 in feces from baseline to V5, and changes from baseline to V4 and V6 in the primary efficacy and all of the secondary efficacy variables. The IPAQ-short physical activity level (total metabolic equivalence of task (MET) minutes per week) was analyzed using a model similar to that used for the primary efficacy variable. The IPAQ total categorical score and the detection of Lpc-37 in feces were presented descriptively. The changes from baseline to V4 and V6 in the primary and secondary efficacy variables were estimated from the models described above.

For all fecal microbiota diversity metric calculations, samples were normalized by rarefaction to account for even sequence depth. Alpha diversity was calculated according to number of unique ASVs and Faith's phylogenetic diversity (PD) (Faith, 1992). The significance of alpha diversity between study groups was determined using the Kruskal-Wallis test (Kruskal and Wallis, 1952). Subsequent pairwise group comparisons between V3 and V5 were carried out, and treatment groups were subset by visit. Beta diversity (pairwise dissimilarity) was calculated using the weighted UniFrac metric (Lozupone and Knight, 2005), and groups were compared by permutational multivariate ANOVA (PERMANOVA) using the adonis test in the R ‘vegan’ package installed in QIIME2 v. 2020.6 (Anderson, 2001; Oksanen et al., 2018). Differential taxa were tested for the main effect of intervention; models were adjusted for visit, sex, and the random effect of participant, using the R package ANCOM2 (Mandal et al., 2015). The ANCOM2 significance cutoff was set to p < 0.05 and p < 0.1 after false discovery rate (FDR) correction by the Benjamini-Hochberg method.

All AEs were tabulated according to the MedDRA. Absolute values and changes from V2 to V6 in safety-related laboratory variables were summarized by group. The gastrointestinal safety parameters (stool consistency and bowel movement frequency) were reported as daily medians and weekly averages (based on the daily medians) by group. The changes from pre-treatment in weekly BSF score and the weekly median number of bowel movements were analyzed in the same fashion as the primary efficacy variable. In the statistical analysis of stool consistency, the response variable was the weekly average of daily medians. In the statistical analysis of bowel movement frequency, the response variable was the weekly median of the daily bowel movement frequencies.

## 3 Results

### 3.1 Participants and baseline characteristics

**Fig. 2:**
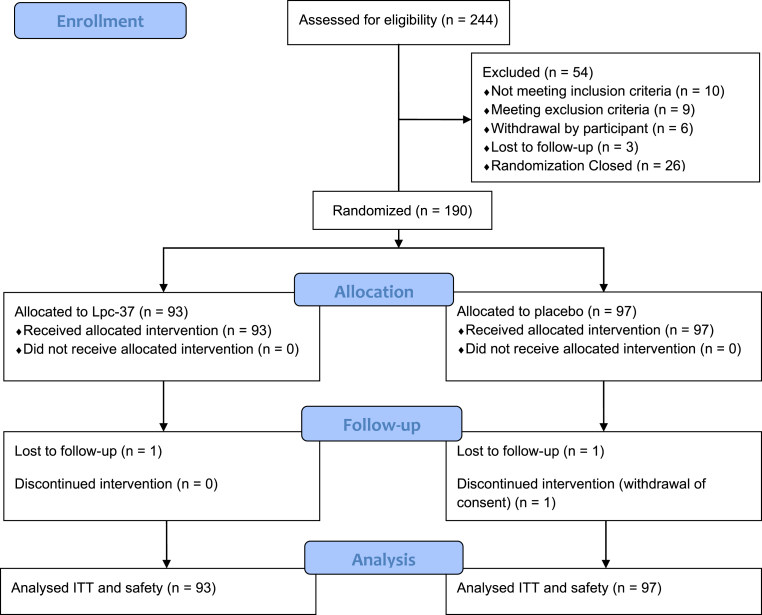
Participant flow in the clinical study. ITT = intention to treat.

A total of 244 participants were screened, of which 190 eligible participants were randomized into the study at V3 (Fig. 2). Demographic and baseline characteristics were similar between groups (Table 1). The mean age of participants was 22.93 ± 2.63 years, 71.1% were White, and 55.3% were female. Overall, 48.9% of participants had high STAI-trait score (>30). Most of the participants (77.9%) were medical students, 34.0% were health science students, and 8.0% were dental students.

**Table 1:** Descriptive statistics of demographic variables (ITT population).

Variable	Class	Lpc-37 (N = 93)	Placebo (N = 97)	Total (N = 190)
Mean age in years (SD)		22.67 (2.56)	23.18 (2.69)	22.93 (2.63)
Mean height in cm (SD)		172.01 (9.71)	171.33 (8.73)	171.66 (9.20)
Mean weight in kg (SD)		68.12 (10.64)	68.95 (10.78)	68.54 (10.69)
Mean BMI in kg/m^2^ (SD)		22.96 (2.51)	23.43 (2.84)	23.20 (2.69)
Sex, n (%)	Female	52 (55.9)	53 (54.6)	105 (55.3)
	Male	41 (44.1)	44 (45.4)	85 (44.7)
STAI-trait score^[1]^, n (%)	High	48 (51.6)	45 (46.4)	93 (48.9)
	Low	45 (48.4)	52 (53.6)	97 (51.1)
STAI-state total score at baseline V3, mean (SD)		29.71 (7.86)	29.56 (8.44)	29.63 (8.14)
Race, n (%)	Asian	23 (24.7)	20 (20.6)	43 (22.6)
	Black or African-American	3 (3.2)	6 (6.2)	9 (4.7)
	Mixed Caucasian and Asian	1 (1.1)	0 (0.0)	1 (0.5)
	Mixed: White/Black	1 (1.1)	0 (0.0)	1 (0.5)
	North African	1 (1.1)	0 (0.0)	1 (0.5)
	White	64 (68.8)	71 (73.2)	135 (71.1)
Ethnicity, n (%)	Hispanic or Latino	1 (1.1)	1 (1.0)	2 (1.1)
	Not Hispanic or Latino	92 (98.9)	96 (99.0)	188 (98.9)
Smoking status, n (%)	Current Daily Smoker	4 (4.3)	4 (4.1)	8 (4.2)
	Current Nondaily Smoker	4 (4.3)	3 (3.1)	7 (3.7)
	Former Smoker	2 (2.2)	5 (5.2)	7 (3.7)
	Never Smoker	83 (89.2)	85 (87.6)	168 (88.4)
University course, n (%)	Dental	5 (5.4)	3 (3.1)	8 (4.2)
	Health Science	14 (15.1)	20 (20.6)	34 (17.9)
	Medical	74 (79.6)	74 (76.3)	148 (77.9)
Academic year, n (%)	1	2 (2.2)	1 (1.0)	3 (1.6)
	2	21 (22.6)	21 (21.6)	42 (22.1)
	3	18 (19.4)	19 (19.6)	37 (19.5)
	4	22 (23.7)	28 (28.9)	50 (26.3)
	5	24 (25.8)	20 (20.6)	44 (23.2)
	6	6 (6.5)	8 (8.2)	14 (7.4)
Type of examination	Practical	4 (4.3)	3 (3.1)	7 (3.7)
	Written	80 (86.0)	83 (85.6)	163 (85.8)
	Written + Practical	9 (9.7)	11 (11.3)	20 (10.5)

Abbreviations: BMI = body mass index, SD = standard deviation, STAI = State Trait Anxiety Inventory. [1] Cutoff point was 30, the median STAI-trait score at baseline.

### 3.2 IP compliance and stability

At the end of the study, IP compliance was 97.6 ± 8.0% in the Lpc-37 group and 98.1 ± 7.4% in the placebo group. Across the seven clinical sites, the average CFU per Lpc-37 capsule was 1.56 × 10^10^ at the start of the study and 1.35 × 10^10^ at the end of the study. The bacterial CFU per placebo capsule remained under the specified maximum of 1.00 × 10^5^.

### 3.3 Primary outcome, STAI-state

**Fig. 3:**
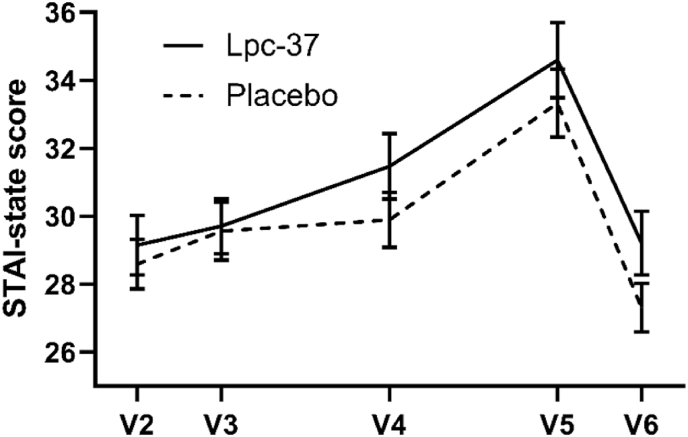
Mean STAI-state score, with standard error, in the Lpc-37 (solid line) and placebo (dashed line) groups at each visit. Note: V3 is baseline, and V5 is the visit after examination.

STAI-state score increased from baseline to V5 in both groups, by 4.77 points in the Lpc-37 group and by 3.77 points in the placebo group (Fig. 3, Supplementary Table S1). At the post-exam visit (V6), the average values decreased in both groups (Fig. 3). Lpc-37 had no statistically significant effect on STAI-state in response to examination stress, compared to placebo. The estimated differences in the STAI-state score between groups was 1.03 (95% CI: -1.62, 3.67; p = 0.446) at V5, 1.37 (95% CI: -0.49, 3.22; p = 0.148) at V4, and 1.85 (95% CI: -0.31, 4.00; p = 0.092) at V6.

In the subgroup analysis based on gender or STAI-trait at baseline, no differences were detected between the Lpc-37 and placebo groups at V5 (p = 0.446 for gender and p = 0.487 for STAI-trait). However, the main effects were significant; STAI-state score increased more in females than in males (estimated difference 1.84, 95% CI: 0.15, 3.53; p = 0.033). Also, the subgroup with high baseline STAI-trait scores had a 2.38 point larger change from baseline over the three visits (95% CI: 0.28, 4.47; p = 0.026) than the subgroup with low baseline STAI-trait scores.

### 3.4 Secondary and ancillary outcomes

#### 3.4.1 Cortisol awakening response (CAR) and evening cortisol

**Fig. 4:**
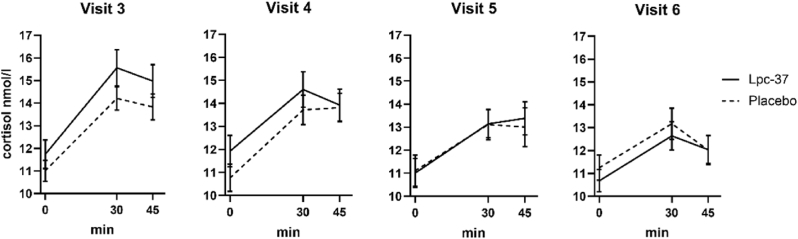
Cortisol awakening response (CAR). Mean cortisol concentration in saliva (nmol/l) over 45 min at each visit in the Lpc-37 (solid line) and placebo (dashed line) groups. Error bars represent standard errors.

In both groups, the CAR declined between visits (Fig. 4). Mean C_max_ values were stable in the placebo group across all visits but declined slightly in the Lpc-37 group (Table 2).

**Table 2:** Descriptive statistics of cortisol awakening response AUC_i_ and C_max_ by group and visit, as mean ± SD (n).

Variable	Visit	Lpc-37 (N = 93)	Placebo (N = 97)	Total (N = 190)
AUC_i,_	visit 3	143.45 ± 155.44 (92)	114.31 ± 121.83 (96)	128.57 ± 139.69 (188)
(nmol/l)*min	visit 4	112.75 ± 129.00 (92)	115.79 ± 132.84(95)	114.29 ± 130.62 (187)
	visit 5	105.72 ± 123.85 (91)	87.80 ± 104.78 (94)	96.61 ± 114.60 (185)
	visit 6	80.06 ± 111.13 (90)	78.29 ± 102.53 (91)	79.17 ± 106.60 (181)

C_max_, nmol/l	visit 3	17.43 ± 7.26 (92)	15.74 ± 5.38 (96)	16.57 ± 6.41 (188)
	visit 4	16.45 ± 7.57 (92)	15.64 ± 6.68 (95)	16.04 ± 7.12 (187)
	visit 5	15.73 ± 7.45 (92)	14.41 ± 6.36 (94)	15.06 ± 6.93 (186)
	visit 6	13.94 ± 5.66 (91)	14.37 ± 6.44 (92)	14.16 ± 6.05 (183)

Large variation was seen in the AUC_i_ for both groups (Table 2). Statistical analyses revealed no significant differences between groups in any of the defined cortisol endpoints at any visits (see Supplementary Table S2 for V5). At V5 the estimated difference in AUC_i_ between groups was 8.06 (95% CI: -25.10, 41.23; p = 0.632). This non-significant result at the top of the hierarchy halted the formal statistical testing of secondary endpoints; analyses of the remaining 23 secondary endpoints were exploratory only.

#### 3.4.2 Depression, anxiety and Stress Scale (DASS-21)

**Fig. 5:**
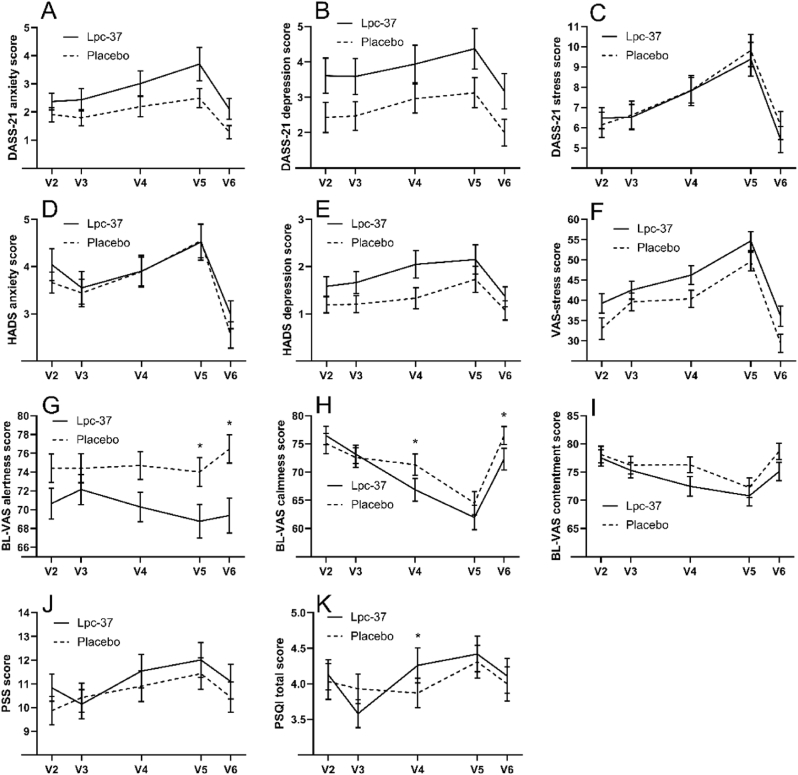
Secondary outcome questionnaire mean scores, with standard errors, in Lpc-37 (solid line) and placebo (dashed line) groups at each visit. Note: V3 is baseline, and V5 is the visit prior to semester examination. A) DASS-21 anxiety score; B) DASS-21 depression score; C) DASS-21 stress score; D) HADS anxiety score; E) HADS depression score; F) VAS-stress score; G) BL-VAS alertness score; H) BL-VAS calmness score; I) BL-VAS contentment score; J) PSS score; K) PSQI total score. * p-value <0.05 for change from baseline between the groups. Abbreviations: BL-VAS = Bond-Lader Visual Analogue Scale; DASS = Depression, Anxiety, Stress Scales; HADS = Hospital Anxiety and Depression Scale; PSQI = Pittsburgh Sleep Quality Index; PSS = Cohen's Perceived Stress Scale; STAI = State Trait Anxiety Inventory; VAS = Visual Analogue Scale, V2 through V6 = visits 1 through 6.

DASS-21 anxiety and depression sub-scores slightly increased towards V5 and declined at V6, resulting in little overall change from baseline (Fig. 5A and B). At V5, the stress sub-score increased from baseline 2.86 points in Lpc-37 group and 3.32 points in placebo group (Fig. 5C). No differences between groups were detected in any of the sub-scores (Supplementary Table S3). Close to half of participants had zero change from baseline in their depression and anxiety scores and due to the abundance of zeros, this outcome was analyzed as a categorical variable with four classes.

#### 3.4.3 Hospital Anxiety and Depression Scale (HADS)

Anxiety and depression levels slightly increased towards V5 and declined to values lower than baseline at V6 for both groups (Fig. 5D and E), but no difference was detected between the groups. Many participants had zero change in HADS depression score. Thus, the change in HADS depression score was analyzed as a categorical variable with three classes (Supplementary Table S3).

#### 3.4.4 Perceived stress (visual analogue scale (VAS)-stress)

In both groups, perceived stress increased towards V5, when the students were approaching the semester examinations, and declined afterwards (Fig. 5F). There was no significant difference between the Lpc-37 and placebo groups at any visit (Supplementary Table S3).

#### 3.4.5 Bond-Lader Visual Analogue Scale (BL-VAS)

In the placebo group, alertness was stable until the examination and increased afterwards (Fig. 5G). In the Lpc-37 group, alertness declined until the examination and increased slightly afterwards. Calmness and contentment scores declined until the examination and increased afterwards for both groups (Fig. 5H and I). Just before the examination (V5), there was no difference between the groups in the calmness score or the contentment score. However, the calmness score was lower in the Lpc-37 group compared with the placebo group at V4 (−4.73; 95% CI: -9.30, −0.17; p = 0.042) and at V6 (−4.64; 95% CI: -9.09, −0.20; p = 0.041). Alertness declined more in the Lpc-37 group compared with the placebo group at V5 (−3.97; 95% CI: -7.78, −0.15; p = 0.042) and at V6 (−6.10; 95% CI: -10.42, −1.79; p = 0.006).

#### 3.4.6 Cohen's Perceived Stress Scale (PSS)

Average changes from baseline in PSS total score were small in both groups (Fig. 5J). In the Lpc-37 group, at all post-randomization visits, PSS total score increased/decreased by about one unit more than it did for the placebo group. However, these differences were not statistically significant (see Supplementary Table S3).

#### 3.4.7 Pittsburgh Sleep Quality Index (PSQI)

**Fig. 6:**
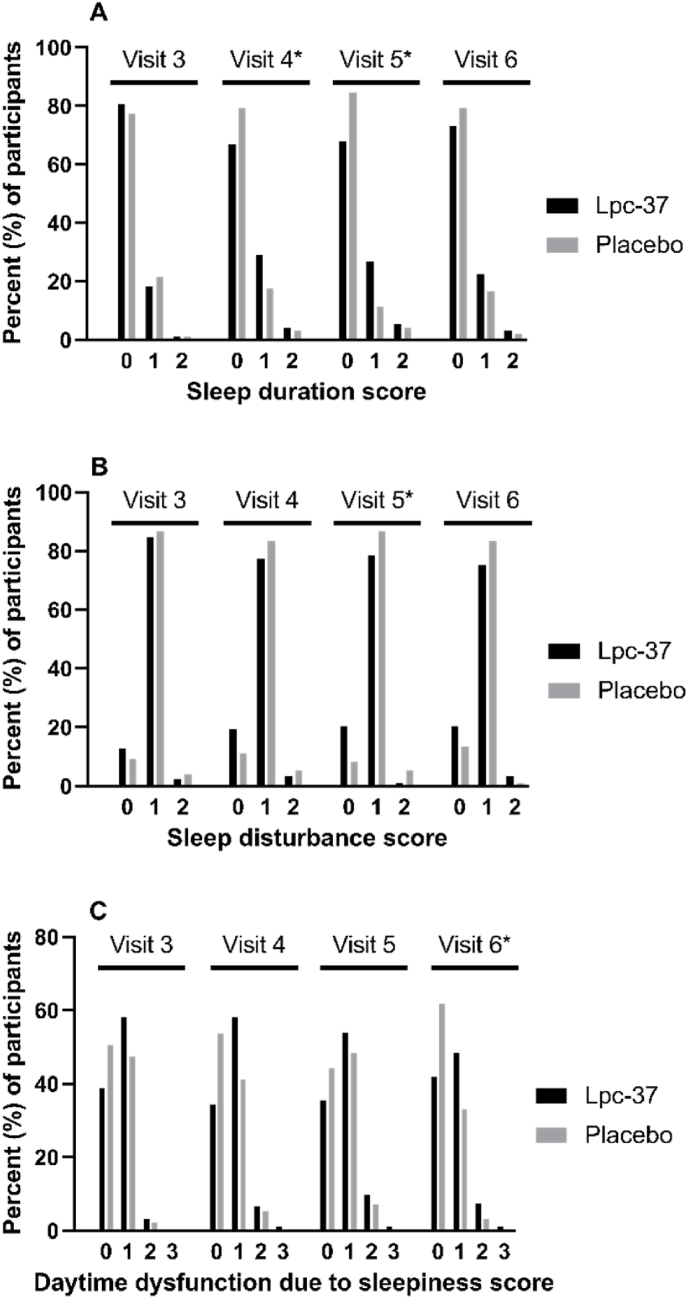
Percent of participants with PSQI sub-scores, in Lpc-37 (black bars) and placebo (grey bars) groups at each visit, for A) sleep duration, B) sleep disturbance, and C) daytime dysfunction due to sleepiness. * p-value <0.05 for odds ratio of change from baseline between the groups.

Changes from baseline in the average PSQI total scores were small in both groups (Fig. 5K). The mean PSQI total score remained under 5, indicating overall good sleep quality. At V5 and V6, PSQI total score did not differ between the Lpc-37 and placebo groups. At V4, the Lpc-37 group had a higher PSQI total score than the placebo group (0.34; 95% CI: 0.10, 0.59; p = 0.007). Changes from baseline in the PSQI sub-scores were also small. At V5, 22.6% of the Lpc-37 group reported shorter sleep duration than at baseline, whereas only 8.2% of the placebo group did so (Fig. 6A). Conversely, 15.1% of the Lpc-37 group experienced less sleep disturbance at V5 compared with baseline, but only 4.1% of the placebo group (Fig. 6B). At V6, in Lpc-37 group 14.0% of participants had a better daytime dysfunction due to sleepiness score than at baseline compared with 22.7% of the participants in placebo group (Fig. 6C).

Results of the statistical analyses for the PSQI sub-scores are presented in Supplementary Table S3. For the duration of sleep score, the odds of an increase from baseline (i.e., less sleep) were higher in the Lpc-37 group compared with placebo group (odds ratio 3.52; 95% CI: 1.46, 8.58; p = 0.005) at V5. The odds of an increase in sleep disturbance score (i.e., more sleep disturbance) from baseline were smaller for the Lpc-37 group than for the placebo group (odds ratio 0.30; 95% CI: 0.11, 0.82; p = 0.020) at V5. In daytime dysfunction due to sleepiness score, the groups did not differ significantly at V5 and V4, but at V6 the placebo group had less daytime dysfunction due to sleepiness compared with Lpc-37 group (odds ratio 2.88; 95% CI: 1.27, 6.49; p = 0.011).

#### 3.4.8 IPAQ-short

According to IPAQ total MET-minutes per week, the participants in both groups had a similar physical activity level at baseline (Lpc-37 mean 3255.27 ± 2176.99, placebo mean 3360.58 ± 2134.59). The MET-minutes decreased from baseline to V5 by −261.12 in Lpc-37 group and −411.82 in placebo group. Most of the participants had a high or moderate IPAQ score at baseline and throughout the study (data not shown). No statistically significant differences in the physical activity levels were detected between the groups.

#### 3.4.9 Fecal microbiota

**Fig. 7:**
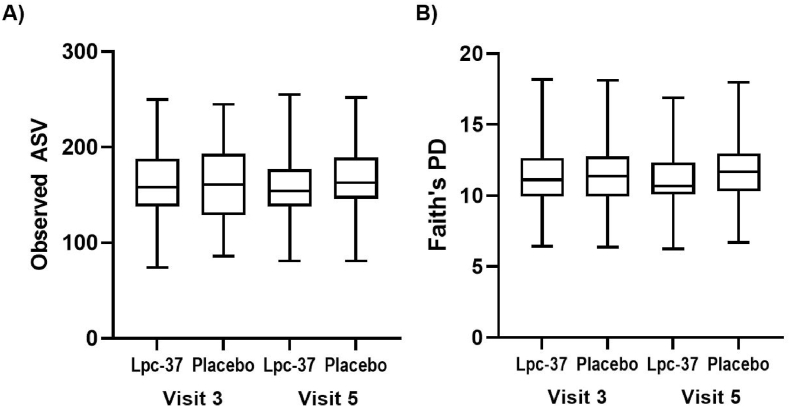
Alpha diversity of Lpc-37 and placebo groups at visit 3 and visit 5, measured using A) observed ASVs and B) Faith's PD. The box represents the 25th to 75th percentiles, and whiskers represent the range of data values.

There were no differences between the study groups for alpha diversity, as measured by observed ASVs and Faith's PD at V3 or at V5 (Fig. 7). We compared the Lpc-37 group versus the placebo group at V3 or at V5. We also compared the alpha diversity at V3 versus V5 within each study group; however, no significant differences were found for any comparisons (p > 0.1). At V3, females in both groups had greater diversity than males (p = 0.071 for Faith's PD).

**Fig. 8:**
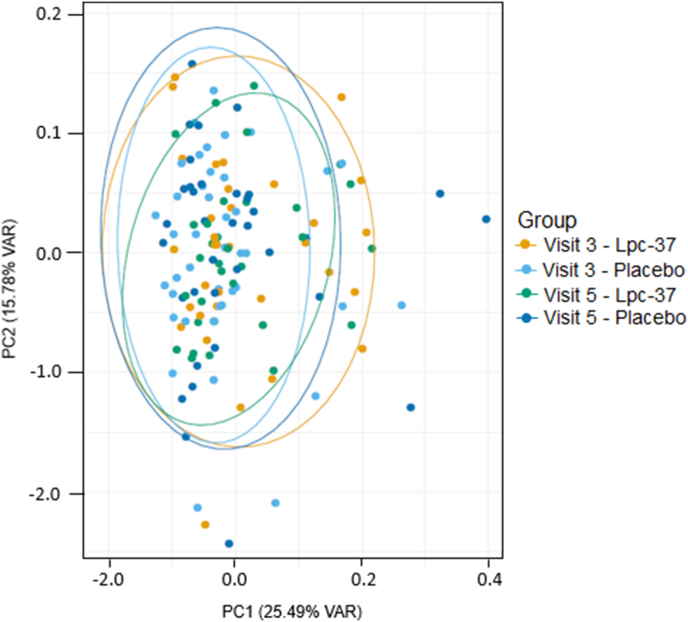
Principal coordinates analysis (PCoA) showing the beta diversity (weighted UniFrac distance) in samples from the Lpc-37 group at V3 (n = 33; orange) and at V5 (n = 33; green) and from the placebo group at V3 (n = 41; light blue) and at V5 (n = 35; dark blue). (For interpretation of the references to colour in this figure legend, the reader is referred to the Web version of this article.)

Beta diversity analyses based on the weighted UniFrac metric indicated the samples did not cluster according to study group or visit (Fig. 8). Beta diversity was investigated separately within the Lpc-37 and placebo groups and by visit at V3 and V5.

The PERMANOVA test showed that within the Lpc-37 and placebo groups, only 7% of the variation in the first principal coordinate (PC1) was due to study factors: participant, site, sex, and visit (Supplementary Table S4). By visit, only 4% of the total variation at V3 and 6% at V5 was explained by study factors: participant, site, sex, and treatment (Supplementary Table S5). At V5, study group (treatment) accounted for less than 1% of the total variation and was not statistically significant (p > 0.1). The PERMANOVA test was significant at V5 for the study factor sex, for the total number of participants (p = 0.027) and within the Lpc-37 (p = 0.023) and placebo groups (p = 0.016). However, R^2^ values revealed that only a small proportion (approximately 3%) of the variance was explained by sex.

The microbiota composition for each group at each visit seemed characteristic to healthy human stool, and no statistically significant differences were seen at phyla, genera or ASV level between groups or timepoints (FDR >0.1) (Supplementary Figs. S2 and S3). As expected, the number of participants with the ASV that represents *L. paracasei* increased after intervention in the Lpc-37 group but not in the placebo group (Supplementary Fig. S1). At V5, 25 of 34 participants in the Lpc-37 group were qPCR-positive for Lpc-37, and 0 of 35 were positive in the placebo group. Lpc-37 supplementation and stress challenge (the examination) did not have a significant effect on fecal microbiota composition.

### 3.5 Safety and vital signs

A total of 318 treatment-emergent adverse events (TEAE) were reported in the study from a total of 117 participants across both groups (Table 3). Of the TEAEs, 163 events were reported in the Lpc-37 group (64.5% of participants, n = 60) and 155 in the placebo group (58.8% of participants, n = 57). No clear differences between the groups could be detected in the incidence of particular types of AE. Headache was the most common AE, reported by 21 (22.6%) participants from the Lpc-37 group and 31 (32%) participants from the placebo group. Forty-eight participants (25.3%) experienced gastrointestinal disorders (32.3% of the Lpc-37 group, 18.6% of the placebo group).

**Table 3:** Summary of adverse events (safety population).

Category	Lpc-37 (N = 93)		Placebo (N = 97)		Total (N = 190)	
	f	n (%)	f	n (%)	f	n (%)
Any AEs	163	60 (64.5)	155	57 (58.8)	318	117 (61.6)
Product-related AEs^[1]^	20	10 (10.8)	20	6 (6.2)	40	16 (8.4)
Severe AEs	12	9 (9.7)	6	5 (5.2)	18	14 (7.4)
Serious AEs^[2]^	1	1 (1.1)	0	0 (0.0)	1	1 (1.1)
AEs leading to study discontinuation	0	0 (0.0)	0	0 (0.0)	0	0 (0.0)
AEs leading to death	0	0 (0.0)	0	0 (0.0)	0	0 (0.0)

Abbreviations: AE = adverse event; n = number of participants with at least one adverse event; N = number of participants in the group(s); f = number of adverse events.

Note: Percentages were calculated as n/N.

^[1]^ Events were classified as product related if they were possibly or probably related to the study product.

^[2]^ Not related to the study product.

Eighteen of the AEs from 14 participants were defined as severe, 12 events in the Lpc-37 group, and 6 in the placebo group. Two of the 18 severe AEs, abdominal discomfort, and nausea, were identified as possibly related to IP, both were from the same participant in the Lpc-37 group. One serious AE (a septic wound infection) not related to the IP was reported in the Lpc-37 group.

Mean systolic blood pressure, diastolic blood pressure, and heart rate values were within normal range at V2 and V6 and remained stable in both groups (data not shown). No clinically meaningful changes were observed for any safety-related laboratory parameters in either group. All participants had one or more out-of-range values for laboratory parameters, but these were minor in all cases and considered to have no clinical implications. The average concentration of urea in urine increased by 101 mmol/L in the placebo group but stayed nearly stable (increased by 4 mmol/L) in the Lpc-37 group. Stool consistency and bowel movement frequency were stable in both groups, and no indication of any differences between the groups was detected (data not shown).

## 4 Discussion

In a meta-analysis of 22 preclinical studies, the administration of probiotics was shown to significantly reduce anxiety-like behavior in rodents, when compared with placebo (Reis et al., 2018). The promising pre-clinical results have only partially translated to human clinical findings; one meta-analysis has found significant reduction in anxiety in humans (McKean et al., 2017), and others have not (Chao et al., 2020; Reis et al., 2018). In this study, the probiotic Lpc-37, which reduces anxiety-like behavior in mice (Stenman et al., 2020), did not reduce stress and anxiety, measured by STAI-state questionnaire, in healthy students facing examination stress in the UK and Ireland.

A recent meta-analysis (Chao et al., 2020) that assessed 10 randomized controlled clinical trials published in the past five years concluded that probiotics are significantly more effective in treating depression than anxiety, and more effective in treating patients with depression and anxiety than healthy individuals under stress. These findings may explain why, in the current study, the primary objective of assessing state anxiety was not met in a stressed, but otherwise healthy and young, population.

Sample size was calculated using a 6-point difference in STAI-state total score between the Lpc-37 and placebo groups at V5. An average reduction of 6 points at V5 between groups was chosen as clinically meaningful, assuming an approximate 16-point increase in STAI-state total score from baseline to V5, based on previous studies (Kato-Kataoka et al., 2016b; Marcos et al., 2004; Nishida et al., 2017; Takada et al., 2016, 2017). However, the current study participants did not experience as much stress as expected before the examination. The anxiety levels in our study were comparable to other European student populations. For instance, in one study on 185 nursing students the mean STAI-state was 32.49 (10.75 SD) (Lavedán Santamaría et al., 2022), while another study on 1021 students facing selective examination reported a mean of 31.53 (11.7 SD) (Fernández-Castillo, 2021). Asian populations seem to have a more elevated STAI-state under comparable circumstances. One study on 637 Japanese medical students reported a mean STAI-state score of 42 (39–46 interquartile range) (Tanifuji et al., 2022). In fact, previous studies conducted in Japan and India showing efficacy of probiotics have reported STAI-state scores above 40 at baseline, and the participants included also reported a smaller variation in this parameter (Nishida et al., 2017; Venkataraman et al., 2021). We hypothesize that a high STAI-state score at baseline and a lower variability among participants may favor detecting differences between groups in similar study designs as ours. In our study, with an increase of 4.77 points in the Lpc-37 group and 3.77 points in the placebo group, it was difficult to show a 6-point difference between groups. Two previous studies (Kato-Kataoka et al., 2016b; Takada et al., 2016), which had comparable study populations (healthy medical students), duration of treatment (8 weeks), use of a placebo, and main outcome; these studies, too, were unable to demonstrate a significant effect on STAI-state of a probiotic intervention—in their case, milk fermented with *Lacticaseibacillus casei* strain Shirota.

Within subgroups based on sex or STAI-trait scores, the two groups did not differ in the primary endpoint. However, regardless of the treatment and relative to males, females experienced a greater change from baseline STAI-state score over the three on-treatment visits. This trend is consistent with the findings of a previous study of *Lactobacillus gasseri* CP2305 in Japanese medical students, with a treatment duration of 12 weeks (Nishida et al., 2017). The change from baseline over the three visits in STAI-state score was larger in participants with high STAI-trait than in participants with low STAI-trait. This relationship was logical since trait anxiety refers to the relatively stable proneness to anxiety. It can be thought of as the tendency to perceive stressful situations as dangerous or threatening and to respond with more intense state anxiety. Thus, the stronger the trait anxiety, the more likely the individual will feel stress before examinations.

Lpc-37 did not affect the cortisol levels of the participants; across all participants, cortisol levels (CAR and evening cortisol) declined over the visits. By contrast, in two previous studies with daily sampling, the placebo group experienced a significant increase in cortisol levels before an examination, but the probiotic group did not. However, these studies, too, found no significant difference between the treatment groups (Kato-Kataoka et al., 2016a, 2016b). Results pooled from three studies (Takada et al., 2016) also showed that changes in cortisol levels were significantly smaller after 8 weeks of probiotic treatment than in placebo groups, but the differences were not significant in individual studies.

The current study did not meet its secondary objectives to demonstrate effects of Lpc-37 over placebo on VAS-stress, DASS-21, HADS, BL-VAS, PSS, and PSQI scores. After the first test in the hierarchical analysis (CAR-AUC_i_) was not statistically significant, any apparent significance among the remaining secondary comparisons would require further confirmation. Multiplicity adjustments, including hierarchical testing, were employed to avoid potential false positives (Type I error) in the assessment of secondary outcomes (Li et al., 2017). Other similar studies on probiotics and stress have not reported using multiplicity adjustments (Kato-Kataoka et al., 2016b; Marcos et al., 2004; Nishida et al., 2017; Takada et al., 2016, 2017).

Results regarding VAS-stress were inconsistent with results of a previous study of milk fermented with *Lacticaseibacillus casei* strain Shirota (Kato-Kataoka et al., 2016b), where the treatment significantly lowered VAS-stress scores compared with those of the placebo after 7–8 weeks of intervention. However, in that study, the students experienced more stress before the examination compared with the present study. In another similar study, 5-weeks of Lpc-37 intake reduced perceived stress compared with placebo (p = 0.048) (Patterson et al., 2020). Baseline PSS scores in the Patterson et al. study were higher than in the current study (placebo 20.72 ± 7.97, Lpc-37 21.89 ± 7.90). In the placebo group, PSS scores increased over the observation period, whereas in the Lpc-37 group, PSS scores decreased (Patterson et al., 2020). It is not clear why the young German participants in that study had PSS scores almost 10 points higher than what we observed in this study. In general, PSS scores are affected by many factors such as sleep (Lee et al., 2013), physical activity (Allesøe et al., 2021) and diet (Khaled et al., 2020). Patterson et al. stratified participants into high and low chronic stress subgroups at screening, using the Trier Inventory for Chronic Stress (TICS) (Schulz et al., 2004), but stated that the mean scores for TICS were in a relatively normal range, even for the subgroup with high chronic stress. A study of the probiotic *Bifidobacterium longum* 1714 during examination stress in university students in Ireland had similar PSS scores to our study and found no difference between treatment and placebo groups (Moloney et al., 2021). *B. longum* 1714 has had a positive effect on daily stress and stress response and has been observed to modulate brain activity patterns in healthy humans (Allen et al., 2016; Wang et al., 2019).

In the current study, baseline DASS-21 anxiety, depression, and stress scores were within the normal range in both groups, but the mean scores for anxiety and depression were higher in Lpc-37 than placebo group across all visits. Higher baseline scores in the Lpc-37 group were also seen in VAS-stress, whereas for BL-VAS alertness the placebo group had a higher baseline score. The HADS anxiety and depression scores were within normal ranges in both groups, and no beneficial effects of Lpc-37 over placebo on anxiety or depression were seen. As in the current study, Moloney et al. found no significant effect on HADS anxiety or depression in their examination stress study with *B. longum* 1714 (Moloney et al., 2021). One previous study detected a significant effect of a probiotic formulation of *Lactobacillus helveticus* R0052 plus *Bifidobacterium longum* R0175 on HADS global score and HADS-anxiety (Messaoudi et al., 2011).

Stress affects sleep quality, and sleep is intertwined with other aspects of mental health (Baglioni et al., 2016; Scott et al., 2021). Medical students often have poorer sleep quality than the general population (Azad et al., 2015), but the ChillEx population had good sleep quality, as evidenced by the mean PSQI total score of ≤5. It was of interest that the placebo group seemed to have a longer duration of sleep but more sleep disturbance at V5, compared with the Lpc-37 group. Sleep disruption is detrimental to mood (Finan et al., 2015), and the quality of sleep seems to be more important than the quantity (Seow et al., 2020). Others have observed that Lpc-37 increased sleep-related recovery and perceived productivity in participants with high chronic stress compared with placebo (Patterson et al., 2020). Whether the shorter sleep duration in the Lpc-37 group relates to the lower BL-VAS alertness and calmness scores in the ChillEx study requires further investigation. By contrast, in another study, the *B. longum* 1714 strain increased sleep duration during exam stress (Moloney et al., 2021) and a recent meta-analysis showed that supplementation with probiotics or paraprobiotics improved sleep quality, reducing PSQI global score by 0.78 points (p < 0.001) (Irwin et al., 2020). However, the meta-analysis did not find a significant result in other subjective sleep scales or in objective sleep parameters measured using polysomnography or actigraphy. This may justify the usage of PSQI as a sensitive marker of sleep quality in probiotic interventions. Interestingly, six out of the nine articles included in the meta-analysis of PSQI scores were conducted with Asian populations. This again begs the question whether the sleep quality of the Asian population is more responsive to probiotic supplementation, perhaps for behavioral, cultural, genetic, or dietary reasons. The clinical relevance of a less than 1-point reduction in the PSQI sleep score in the meta-analysis with probiotic supplementation is uncertain. However, melatonin, which is widely used to treat sleep difficulties, conferred a comparably small 1.24-point reduction in PSQI score, according to a recent meta-analysis (Fatemeh et al., 2022). The healthy students in the ChillEx study did not report sleep problems, leaving no room for improvement in sleep quality. As our results are exploratory and seem to conflict with some of the earlier results with probiotic interventions and sleep, more research is necessary to confirm the findings. Recent studies also argue that microbiota-gut-brain axis may affect sleep but the exact mechanisms are to be discovered (Sen et al., 2021).

The current study found no measurable changes in fecal microbiota diversity or composition in response to treatment or to examination stress. There appeared to be a small effect of sex for both microbiota diversity and composition that was independent of treatment or visit. Gender-specific differences in the gut microbiome composition have also been noted in other clinical studies (reviewed by Kim et al., 2020); they were not investigated further here. Two post-intervention measures in the Lpc-37 group—the increased abundance of the ASV corresponding to *L. paracasei* and the mostly Lpc-37-positive qPCR results—indicated that the participants were compliant in consumption of the study products. Thus, reduced adherence to the study product most likely does not explain the lack of efficacy.

The main limitation of this trial was that the population studied did not increase STAI-state before examination as predicted in the study design. Another limitation was that the protocol did not include analysis of potential metabolites derived from the probiotic in blood, urine, or feces that could affect cognitive and mood function. It is possible that although the fecal microbiota composition did not differ between the study groups, the functionality of the microbiota might have shown changes. Also, it is plausible that the study design was not stringent enough on the use of possible co-founders, such as alcohol consumption and other potential co-founders in the diet that could have affected the perception of stress in university students facing examination. Thus, it is recommended that future studies with probiotics on students facing examination include sufficiently stressed populations (e.g. a baseline STAI-state score above 40) and have a stricter control of co-founders. In our study, Lpc-37 did not affect stress and anxiety in a student population facing examinations, and the secondary analyses yielded no significant results, when corrected for multiplicity. This study contributes data to the psychobiotic literature and calls for careful study designs to translate the promising animal study results to future clinical studies.

## Funding

The clinical trial was funded by Danisco Sweeteners Oy.

## Declaration of competing interest

The authors declare the following financial interests/personal relationships which may be considered as potential competing interests: Sanna M. Mäkelä, Jenni Reimari, Kara C. Evans, Ashley A. Hibberd, Nicolas Yeung, and Alvin Ibarra are employees of International Flavors & Fragrances (IFF), which manufactures and sells the investigational product, Lpc-37. Síle Griffin and Elaine Patterson are former employees of IFF. No other conflicts of interest are reported by authors.

The clinical trial was funded by Danisco Sweeteners Oy, a legal entity of IFF.

## Data Availability

The authors do not have permission to share data.
