# Supplementary material for: Efficacy and safety of Lacticaseibacillus paracasei Lpc-37® in students facing examination stress: A randomized, triple-blind, placebo-controlled clinical trial (the ChillEx study)
Source: Brain Behav Immun Health. 2023 Aug 1;32:100673. doi: 10.1016/j.bbih.2023.100673 (PMC10474370; doi:10.1016/j.bbih.2023.100673)
Supplement: Multimedia component 1 [file mmc1.docx]

# Efficacy and safety of *Lacticaseibacillus paracasei* Lpc-37® in Students Facing Examination Stress: A Randomized, Triple-Blind, Placebo-Controlled Clinical Trial (the ChillEx study)

Sanna M. Mäkelä^a^, Síle M. Griffin^a^, Jenni Reimari^a^, Kara C. Evans^b^, Ashley A. Hibberd^b^, Nicolas Yeung^a^, Alvin Ibarra^a^, Jouni Junnila^c,1^, Jari Turunen^c^, Ronnie Beboso^d^, Balgit Chhokar^e^, Timothy G. Dinan^f^, John Cryan^f^, Elaine Patterson^a^

Affiliations:

^a^IFF Health & Biosciences, Kantvik, Finland

^b^IFF Health & Biosciences, Madison, USA

^c^4Pharma Ltd, Turku, Finland

^d^MeDiNova North London Dedicated Research Center, London, UK

^e^MeDiNova East London Dedicated Research Center, London, UK

^f^APC Microbiome Ireland, University College Cork, Cork, Ireland

^1^Present address: EstiMates Oy, Espoo, Finland

Corresponding author: Sanna M. Mäkelä

# Supplementary methods

### Inclusion criteria

In order to participate in this study, participants had to meet all of the following inclusion criteria:

1. Voluntary, written, informed consent to participate in the study.
2. Enrolled in medical, dental or health science university/institute of technology course with intention to sit for semester examination(s).
3. Male or female, aged between 18 to 40 years (inclusive).
4. BMI between 18.5 to 29.9 kg/m2.
5. Blood, urine and gastrointestinal safety parameters and blood pressure measurement at Baseline to indicate they were healthy in the opinion of the PI.
6. In good general health as estimated by the PI, based on medical history (self-reported).
7. Ability of the participant (in the PIs opinion) to comprehend the full nature and purpose of the study including possible risks and side effects.
8. Ability of the participant (in the PIs opinion) to fully comprehend and self-complete all participant reported outcomes (in UK English language).
9. Willing to maintain habitual diet (including caffeine and alcohol), physical activity patterns and smoking habits throughout the intervention period.
10. Agreement to comply with the protocol and study restrictions.
11. Available for all study visits.
12. Females of child-bearing potential required to provide a negative urine pregnancy test and be using effective contraception (e.g. surgically sterilized [tubal ligation or hysterectomy or partner was vasectomized, with sterility confirmed]) or use an intrauterine device, a diaphragm or condom combined with contraceptive sponge, foam or jelly or be using an oral contraceptive for at least 2 cycles before the screening visit (visit 2). Women who were in same sex relationships or abstaining from sex were not required to take a pregnancy test or use effective contraception.
13. Male participants agreed to use a condom during sexual intercourse from visit 3 onwards.
14. Covered by health insurance system and / or in compliance with the recommendations of National Law in force relating to biomedical research.

### Exclusion criteria

In order to participate in this study, the participants did not meet any of the following exclusion criteria:

1. Suspected diagnosis of one or more Diagnostic and Statistical Manual of Mental Disorders, 4^th^ edition (DSM-IV) axis 1 disorder(s), including but not limited to current major depression, anxiety disorder, bipolar spectrum disorder or schizophrenia, as determined by MINI and/or currently diagnosed with one or more DSM-IV axis 1 disorder(s), per DSM-IV.
2. Had a significant acute or chronic coexisting illness (cardiovascular, gastrointestinal [irritable bowel syndrome or inflammatory bowel disease], immunological, metabolic [including diabetes and cardiovascular disease]), neurodevelopmental or any condition which contraindicated, in the PIs judgement, entry to the study.
3. Had been taking (from visit 1 onwards) or had previously taken (last 4 weeks prior to visit 1) psychoactive medication (anxiolytics, sedatives, hypnotics, anti-psychotics, anti‑depressants, anti-convulsants, centrally acting corticosteroids, opioid pain relievers).
4. Had been taking (from visit 1 onwards) medication or dietary supplements that the PI believed would interfere with the objectives of the study, pose a safety risk or confound the interpretation of the study results (e.g. melatonin, St. John’s Wort, Rescue^®^ products including Rescue Remedy^®^, Rescue^®^ Energy, Rescue^®^ Pastilles, Rescue^®^ Pearls, Rescue Sleep^®^ Rescue Plus™, omega-3 dietary supplements (including fish oil), Cannabidiol, non-steroidal anti‑inflammatory drugs, over the counter (OTC) sleep medication (not categorized as sedatives, hypnotics or anti‑depressants), anti‑coagulants, anti-cholinergic drugs or acetylcholinesterase inhibitors, proton pump inhibitors, anti-histamines that cause drowsiness, pseudoephedrine).
5. Recent (within last 4 weeks prior to visit 1) or ongoing antibiotic therapy.
6. Had been taking (from visit 1 onwards) concentrated sources of probiotics and/or prebiotics other than the provided study products (e.g. probiotic/prebiotic tablets, capsules, drops or powders), including yoghurts/yoghurt drinks.
7. Pregnant or lactating female or pregnancy planned during the intervention period.
8. Had self-reported dyslexia.
9. History of or current alcohol, drug or medication abuse (self-reported).
10. Self-declared illicit drug users (including cannabis and cocaine) in the past 1 month prior to visit 1.
11. Excessive alcohol consumption (consuming >8 units of alcohol for men and >6 units of alcohol for women in a single session) >3 times per week for 3 weeks prior to visit 1.
12. Significant change in tobacco, snuff, nicotine and e-cigarette usage habits in the past 1 month before visit 1 or planned cessation of the use of these products during the intervention period.
13. Contraindication to any substance in the IP.
14. Participation in another study with any IP or drug within 60 days of visit 1.
15. The PI believed that the participant may be uncooperative and/or noncompliant and should therefore not participate in the study.
16. Participant under administrative or legal supervision.
17. Previous participation in the ChillEx study.

### Hierarchy of the secondary efficacy endpoints

Twenty-four secondary efficacy endpoints were analyzed in a hierarchical manner using a fixed sequence procedure. Formal testing of the secondary outcomes proceeded, in the following order, until the first null hypothesis was accepted:

1. Change in AUC_i_ (CAR) from baseline (visit 3) to 8 weeks (visit 5)

2. Change in Cmax of cortisol from baseline (visit 3) to 8 weeks (visit 5)

3. Change in cortisol at awakening from baseline (visit 3) to 8 weeks (visit 5)

4. Change in evening cortisol from baseline (visit 3) to 8 weeks (visit 5)

5. Change in perceived stress (VAS-stress) from baseline (visit 3) to 8 weeks (visit 5)

6. Change in DASS-21 depression scale score from baseline (visit 3) to 8 weeks (visit 5)

7. Change in DASS-21 anxiety scale score from baseline (visit 3) to 8 weeks (visit 5)

8. Change in DASS-21 stress scale score from baseline (visit 3) to 8 weeks (visit 5)

9. Change in HADS depression score from baseline (visit 3) to 8 weeks (visit 5)

10. Change in HADS anxiety score from baseline (visit 3) to 8 weeks (visit 5)

11. Change in PSS total score from baseline (visit 3) to 8 weeks (visit 5)

12. Change in BL-VAS alertness score from baseline (visit 3) to 8 weeks (visit 5)

13. Change in BL-VAS contentment score from baseline (visit 3) to 8 weeks (visit 5)

14. Change in BL-VAS calmness score from baseline (visit 3) to 8 weeks (visit 5)

15. Change in PSQI total score from baseline (visit 3) to 8 weeks (visit 5)

16. Change in PSQI duration of sleep score from baseline (visit 3) to 8 weeks (visit 5)

17. Change in PSQI sleep disturbance score from baseline (visit 3) to 8 weeks (visit 5)

18. Change in PSQI sleep latency score from baseline (visit 3) to 8 weeks (visit 5)

19. Change in PSQI daytime dysfunction due to sleepiness score from baseline (visit 3) to 8 weeks (visit 5)

20. Change in PSQI sleep efficiency score from baseline (visit 3) to 8 weeks (visit 5)

21. Change in PSQI subjective sleep quality score from baseline (visit 3) to 8 weeks (visit 5)

22. Change in PSQI use of sleep medication score from baseline (visit 3) to 8 weeks (visit 5)

23. Change in area under the curve with respect to ground (AUC_g_) (CAR) from baseline (visit 3) to 8 weeks (visit 5)

24. Change in cortisol mean increase from baseline (visit 3) to 8 weeks (visit 5)

## 16S sequencing bioinformatic analyses

Sequencing data were analyzed using QIIME2 v. 2020.6 (Bolyen et al., 2019). Only samples from the per-protocol (PP) population were analyzed (n = 144/156; 12 samples were excluded due to ambient conditions during shipment from site to central laboratory). Two samples containing fewer than 10,000 reads were removed from the study. Reads were demultiplexed using QIIME DEMUX, and QIIME DADA2 was used for denoising, dereplication, and removal of chimeras by the consensus method (Callahan et al., 2016). The reverse reads were trimmed at 180 bp and overlapping sequences were paired. Taxonomy was assigned to aligned amplicon sequence variants (ASVs) using q2-feature-classifier (classify-sklearn) trained on the V4 region of sequences in the RDP Classifier (v. 2.13; training set No. 18 July 2020 release) (Pedregosa et al., 2011). Taxa compositions are reported as relative abundance (% of total sequences).

Supplementary Table S1. Descriptive statistics of STAI-state total score by group and visit, as mean ± SD (n).

| **Parameter** | **Visit** | **Lpc-37 (N=93)** | **Placebo (N=97)** | **Total (N=190)** |
| --- | --- | --- | --- | --- |
| STAI-state total score | Visit 2 | 29.15 ± 8.39 (93) | 28.59 ± 7.20 (97) | 28.86 ± 7.79 (190) |
|  | Visit 3 | 29.71 ± 7.86 (93) | 29.56 ± 8.44 (97) | 29.63 ± 8.14 (190) |
|  | Visit 4 | 31.47 ± 9.28 (93) | 29.89 ± 7.87 (96) | 30.67 ± 8.61 (189) |
|  | Visit 5 | 34.59 ± 10.67 (92) | 33.33 ± 9.89 (97) | 33.94 ± 10.27 (189) |
|  | Visit 6 | 29.21 ± 9.00 (92) | 27.31 ± 7.06 (95) | 28.24 ± 8.11 (187) |

Supplementary Table S2. Analysis results for cortisol-related secondary endpoints in Lpc-37 group versus placebo group.

| **Parameter** | **V5 estimated effect** | **95% CI, V5 estimated effect** | **p-value, V5** | **p-value, overall treatment effect** |
| --- | --- | --- | --- | --- |
| AUC_i_ | 8.06 | -25.10, 41.23 | 0.632 | 0.803 |
| C_max_ (nmol/l) | 0.04 | -2.28, 2.35 | 0.974 | 0.560 |
| Cortisol at awakening (nmol/l) | -0.25 | -2.03, 1.53 | 0.781 | 0.920 |
| Evening cortisol (nmol/l) | -0.24 | -1.94, 1.45 | 0.778 | 0.8683 |
| AUC_g_ | -29.24 | -114.10, 55.62 | 0.498 | 0.413 |
| Mean increase | 0.57 | -0.93, 2.07 | 0.455 | 0.931 |

Supplementary Table S3. Estimated differences in Least Square Means or odds ratio (OR) estimates for change from baseline in Lpc-37 group as compared to the placebo group in VAS-stress, DASS-21, HADS, BL-VAS, PSS, PSQI, and IPAQ (ITT population). Statistically significant values are in bold.

| **Parameter** | **Visit 5** | **Visit 4** | **Visit 6** |
| --- | --- | --- | --- |
| VAS-Stress  (estimate; 95% CI; p) | 3.70; -2.29, 9.70; p = 0.224 | 4.67; -0.74, 10.08; p = 0.090 | 5.86; -0.63, 12.34; p = 0.074 |
| DASS-21, anxiety  (OR; 95% CI; p) | 1.41; 0.73, 2.75; p = 0.307 | 1.26; 0.65; 2.45, p = 0.499 | 1.44; 0.72, 2.85; p = 0.302 |
| DASS-21, depression  (OR; 95% CI; p) | 1.44; 0.72, 2.88; p = 0.299 | 1.17; 0.59, 2.34; p = 0.655 | 1.50; 0.73, 3.05; p = 0.268 |
| DASS-21, stress  (estimate; 95% CI; p) | -0.39; -2.28, 1.50; p = 0.686 | -0.06; -1.52, 1.40; p = 0.934 | -0.57; -2.24, 1.11; p = 0.506 |
| HADS, anxiety  (estimate; 95% CI; p) | -0.07; -0.92, 0.79; p = 0.877 | -0.04; -0.66, 0.58; p = 0.899 | 0.44; -0.28, 1.16; p = 0.230 |
| HADS, depression  (OR; 95% CI; p) | 1.54; 0.74, 3.21; p = 0.251 | 1.82; 0.87, 3.80; p = 0.112 | 1.21; 0.57, 2.54; p = 0.623 |
| BL-VAS, alertness (estimate; 95% CI; p) | -3.97; -7.78, -0.15; **p = 0.042** | ‑3.13; -6.51, 0.25; p = 0.069 | ‑6.10; -10.42, -1.79; **p = 0.006** |
| BL-VAS, calmness (estimate; 95% CI; p) | -2.85; -8.05, 2.34; p = 0.280 | -4.73; -9.30, -0.17; **p = 0.042** | -4.64; -9.08, -0.20; **p = 0.041** |
| BL-VAS, contentment (estimate; 95% CI; p) | -0.84; -4.92, 3.23; p = 0.683 | ‑3.21; -6.46, 0.04; p = 0.053 | ‑3.14; -6.90, 0.61; p = 0.101 |
| PSS  (estimate; 95% CI; p) | 0.89; -0.58, 2.37; p = 0.235 | 0.85; -0.37, 2.08; p = 0.172 | 0.97; ‑0.46, 2.41; p = 0.182 |
| PSQI, total score (estimate; 95% CI; p) | 0.18; -0.09, 0.45; p = 0.194 | 0.34; 0.10, 0.59; **p = 0.007** | 0.23; -0.04, 0.50; p = 0.099 |
| PSQI, duration of sleep (OR; 95% CI; p) | 3.52; 1.46, 8.54; **p = 0.005** | 2.44; 1.03, 5.79; **p = 0.044** | 1.83; 0.75, 4.47; p = 0.186 |
| PSQI, sleep disturbance (OR; 95% CI; p) | 0.30; 0.11, 0.82; **p = 0.020** | 0.56; 0.20, 1.53; p = 0.256 | 1.02; 0.39, 2.69; p = 0.972 |
| PSQI, sleep latency  (OR; 95% CI; p) | 1.32; 0.61, 2.85; p = 0.484 | 1.46; 0.67, 3.18; p = 0.343 | 0.93; 0.43, 2.03; p = 0.856 |
| PSQI, daytime dysfunction (OR; 95% CI; p) | 1.42; 0.64, 3.12; p = 0.387 | 2.16; 0.97, 4.80; p = 0.059 | 2.88; 1.27, 6.49; **p = 0.011** |
| PSQI, sleep efficiency  (OR; 95% CI; p) | 1.09, 0.50, 2.36; p = 0.827 | 1.27; 0.58, 2.78; p = 0.547 | 0.90; 0.40, 1.99; p = 0.788 |
| PSQI, subjective sleep quality (OR; 95% CI; p) | 1.38; 0.58, 3.31; p = 0.468 | 2.19; 0.91, 5.28; p = 0.081 | 1.95; 0.81, 4.70; p = 0.136 |
| IPAQ-Short  (estimate; 95% CI; p) | 0.07; -0.17, 0.32; p = 0.554 | 0.09; -0.17, 0.34; p = 0.500 | 0.26; -0.06, 0.58; p = 0.107 |


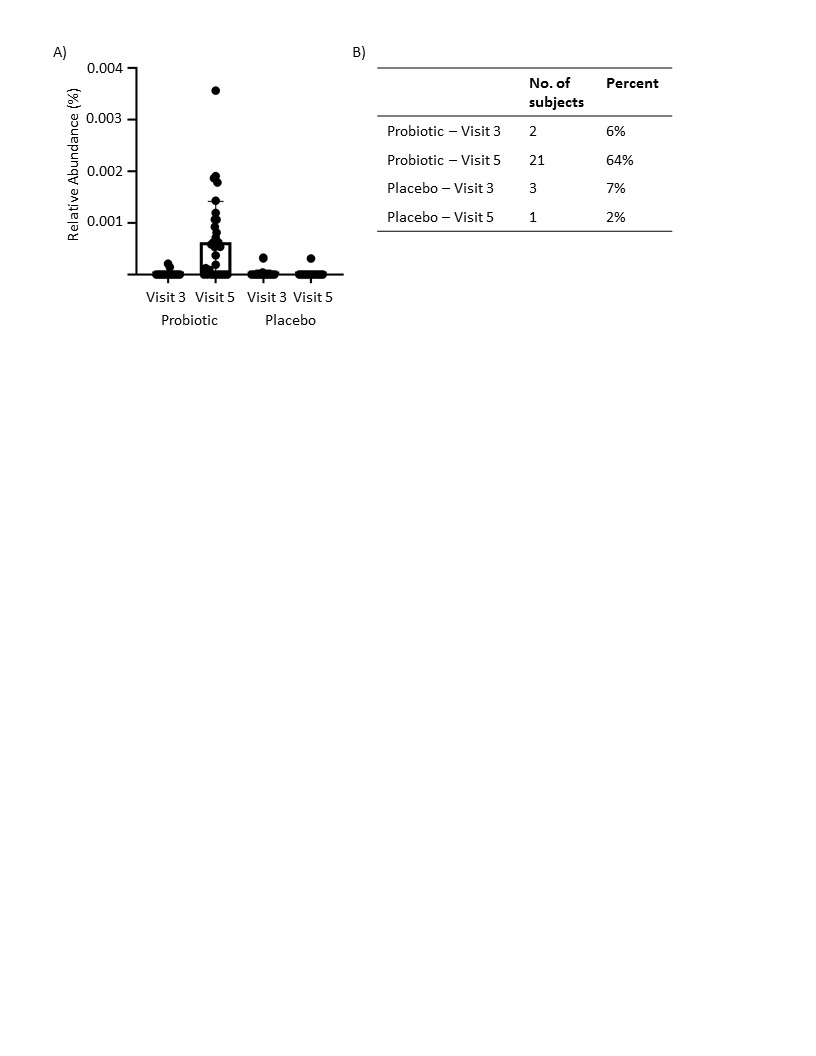


Supplementary Figure S1. The relative abundance (%) of the ASV representing L. paracasei Lpc-37 is shown for each participant at visit 3 and visit 5 for probiotic Lpc-37 and placebo groups. B) The number and percentage of participants who were positive for the ASV representing Lpc-37, by study group (Lpc-37 probiotic or placebo) and visit.


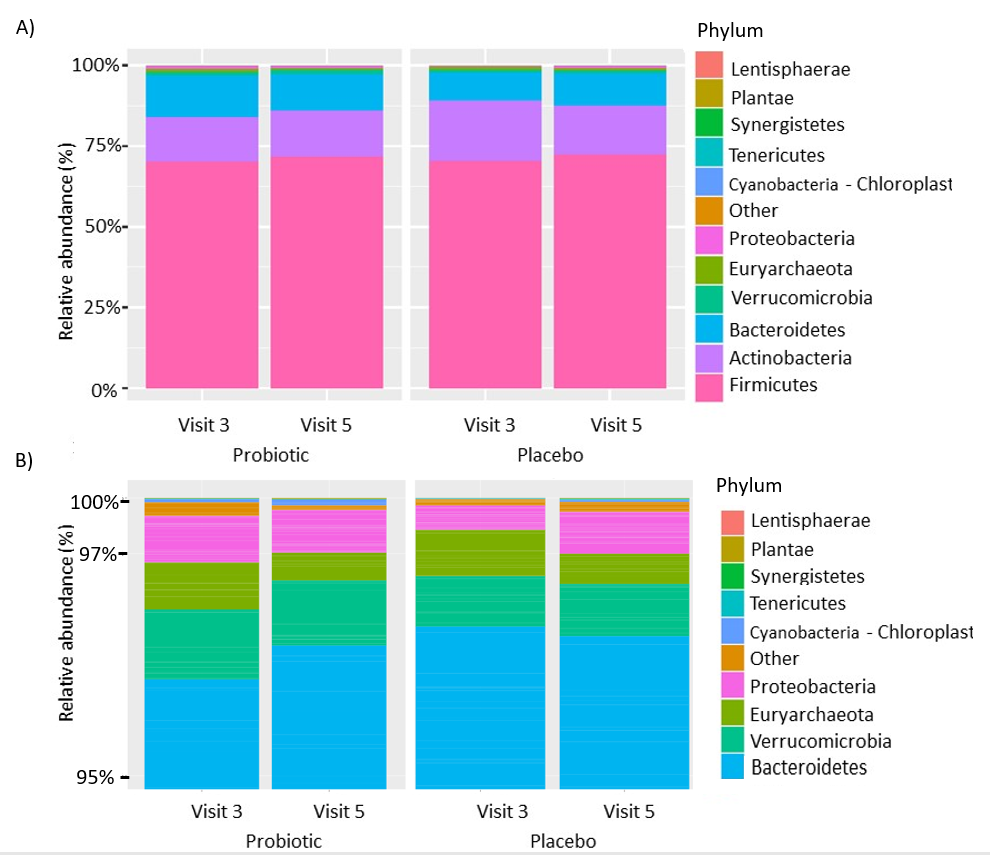


Supplementary Figure S2. A) Phylum-level taxonomic composition of the fecal microbiota from probiotic Lpc-37 and placebo groups at visit 3 (probiotic Lpc-37, n = 33; placebo, n = 41) and visit 5 (probiotic Lpc-37, n = 33; placebo, n = 35). Each phylum is represented by a different colored section in the bar graph. Relative abundance was calculated by summing the ASV counts from each participant for each phylum and then dividing by the total number of counts for each study group. B) An enlargement of the phyla that made up less than 5% of the total phylum ASV counts.


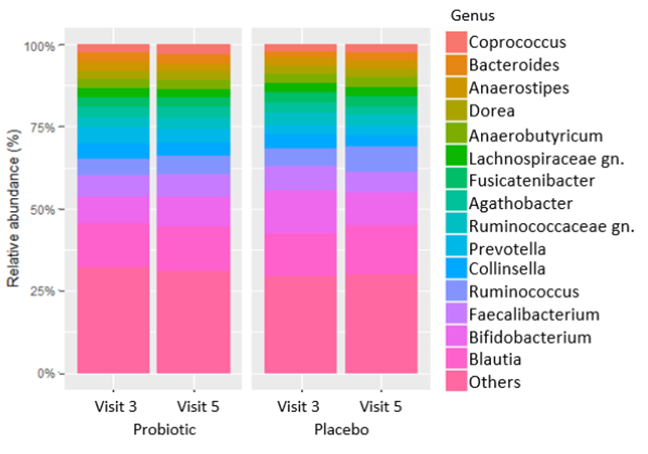


Supplementary Figure S3. Genus-level taxonomic composition of the fecal microbiota from probiotic Lpc-37 and placebo groups at visit 3 (probiotic Lpc-37, n = 33; placebo, n = 41) and visit 5 (probiotic Lpc-37, n = 33; placebo, n = 35). Relative abundance was calculated by summing the ASV counts from each participant for each genus and then dividing by the total number of counts for each study group.

Supplementary Table S4. PERMANOVA (adonis) test of the effect of study factors on beta diversity within the Lpc-37 and placebo groups.

|  | **Lpc-37** | | **Placebo** | |
| --- | --- | --- | --- | --- |
|  | **R^2^** | **P value** | **R^2^** | **P value** |
| **Participant ID** | 0.015 | 0.413 | 0.009 | 0.651 |
| **Site** | 0.018 | 0.277 | 0.011 | 0.544 |
| **Sex** | 0.036 | 0.023 | 0.035 | 0.016 |
| **Visit** | 0.003 | 0.995 | 0.011 | 0.525 |
| **Residuals** | 0.928 | NA | 0.934 | NA |
| **Total** | 1.00 | NA | 1.00 | NA |

Supplementary Table S5. PERMANOVA (adonis) test of the effect of study factors on beta diversity overall across treatment groups at visit 3 and visit 5.

|  | **Visit 3** | | **Visit 5** | |
| --- | --- | --- | --- | --- |
|  | **R^2^** | **p-value** | **R^2^** | **p-value** |
| **Participant ID** | 0.006 | 0.913 | 0.011 | 0.666 |
| **Site** | 0.006 | 0.924 | 0.007 | 0.891 |
| **Sex** | 0.016 | 0.344 | 0.034 | 0.027 |
| **Treatment** | 0.013 | 0.501 | 0.007 | 0.923 |
| **Residuals** | 0.960 | NA | 0.941 | NA |
| **Total** | 1 | NA | 1 | NA |

# References:

Bolyen, E., Rideout, J. R., Dillon, M. R., Bokulich, N. A., Abnet, C. C., Al-Ghalith, G. A., Alexander, H., Alm, E. J., Arumugam, M., Asnicar, F., Bai, Y., Bisanz, J. E., Bittinger, K., Brejnrod, A., Brislawn, C. J., Brown, C. T., Callahan, B. J., Caraballo-Rodríguez, A. M., Chase, J., . . . Caporaso, J. G. (2019). Reproducible, interactive, scalable and extensible microbiome data science using QIIME 2. *Nature Biotechnology*, *37*(8), 852-857. <https://doi.org/10.1038/s41587-019-0209-9>

Callahan, B. J., McMurdie, P. J., Rosen, M. J., Han, A. W., Johnson, A. J. A., & Holmes, S. P. (2016). DADA2: High-resolution sample inference from Illumina amplicon data. *Nature Methods*, *13*(7), 581-583. <https://doi.org/10.1038/nmeth.3869>

Pedregosa, F., Varoquaux, G., Gramfort, A., Michel, V., Thirion, B., Grisel, O., Blondel, M., Prettenhofer, P., Weiss, R., Dubourg, V., Vanderplas, J., Passos, A., Cournapeau, D., Brucher, M., Perrot, M., & Duchesnay, E. (2011). Scikit-learn: Machine Learning in Python. *J Mach Learn Res*, *12*, 2825-2830.
